# Are there intra-operative hemodynamic differences between the Coliseum and closed HIPEC techniques in the treatment of peritoneal metastasis? A retrospective cohort study

**DOI:** 10.1186/s12957-017-1119-2

**Published:** 2017-02-21

**Authors:** Cristina Rodríguez Silva, Francisco Javier Moreno Ruiz, Inmaculada Bellido Estévez, Joaquin Carrasco Campos, Alberto Titos García, Manuel Ruiz López, Ivan González Poveda, Jose Antonio Toval Mata, Santiago Mera Velasco, Julio Santoyo Santoyo

**Affiliations:** Universidad de Málaga, Departamento de Farmacología, Servicio de Cirugía General, Digestiva y Trasplantes, Hospital Regional Universitario de Málaga, Avda Carlos Haya, 29020 Malaga, Spain

**Keywords:** Peritoneal carcinomatosis, Coliseum technique, Closed technique, HIPEC, Peritoneal metastasis

## Abstract

**Background:**

Although two main methods of intraoperative hyperthermic intraperitoneal chemotherapy (HIPEC) are currently accepted, the superiority of one over the other has not yet been demonstrated. The purpose of this study was to determine whether there are hemodynamic and temperature differences between patients who received HIPEC in two different techniques, open versus closed abdomen.

**Methods:**

This retrospective study was conducted in our center between 2011–2015 in 30 patients who underwent surgery for peritoneal carcinomatosis secondary to colorectal cancer, in whom cytoreduction and HIPEC were performed by the Coliseum (15) or closed techniques (15). The main end points were morbidity, mortality, hemodynamic changes, and abdominal temperature. The comparative analysis of quantitative variables at different times was done with the parametric repeated measure ANOVA for those variables that fulfilled the suppositions of normality and independence and the Friedman non-parametric test for the variables that did not fulfill either of these suppositions.

**Results:**

There were no deaths in either group. The incidence of postoperative complications in the Coliseum group was 53% (8 patients), grade II–III. The incidence of complications in the closed group was 13% (2 patients), grade II–III. The intra-operative conditions regarding the systolic and diastolic pressures were more stable using the closed abdomen technique (but not significantly so). We found statistically significant differences in abdominal temperature in favor of the closed technique (*p =* 0.009).

**Conclusions:**

Both HIPEC procedures are similar. In our series, the closed technique resulted in a more stable intra-abdominal temperature.

## Background

In the past, peritoneal metastasis of colorectal carcinoma was considered to be a terminal stage of the disease, associated with a poor prognosis, such that patients were offered supportive care and systemic chemotherapy, with or without palliative surgery [1]. Metastatic disease is the main cause of death in patients with colorectal cancer but, unlike other tumors, its presence in either the abdominal cavity or distant in the liver or lung does not now prevent treatment with a curative intention in selected groups of patients [2]. Since some 20 years ago, an alternative treatment modality has been developed, based on the combination of surgery associated with chemotherapy, such that the macroscopic disease is treated with cytoreductive surgery (CRS) followed by treatment of the residual microscopic disease with intraoperative hyperthermic intraperitoneal chemotherapy (HIPEC) [1]. This integrated procedure has a curative intention and aims to improve the quality of life and increase the rates of survival. HIPEC can be undertaken in several ways, with no clear advantage shown for any one method over the others. The best known of these are the Coliseum technique and the closed technique. Although each HIPEC perfusion technique has its own advantages and inconveniences, no controlled prospective studies have compared the different methods of administration [3]. As stated in Milan in 2006 at a consensus meeting of the Peritoneal Surface Oncology Group International, the debate is still open regarding the best method to perform the HIPEC procedure. Not enough scientific evidence has yet been published to confirm the superiority of one technique over the other [4].

The aim of this study was to assess the differences in the intra-operative parameters during HIPEC administration between the open and the closed techniques, as well as to identify perioperative morbidity and mortality.

## Methods

### Patients

This retrospective cohort study comprised 30 patients with peritoneal metastasis of colorectal origin, who all fulfilled the criteria to undergo CRS and HIPEC at the Regional University Hospital of Malaga, from December 2011 to March 2015. All the patients were assessed, treated, and reviewed by the same surgical team. Most of the patients were originally admitted to our own hospital, with just 3 cases referred from other hospitals in the Malaga area. The study was authorized by the hospital Ethics Committee.

The inclusion criteria for patients to undergo the procedure included the following: an age between 18 and 70 years; patients with peritoneal metastasis with a peritoneal carcinomatosis index (PCI) ≤26; achievement of macroscopically complete surgical cytoreduction or with tumor remains no greater than 2.5 mm, with a completeness of cytoreduction score (CC score) of 0 or 1; patients with a life expectancy greater than 12 weeks and with a performance status (Eastern Cooperative Oncology Group) ≤2; patients with no extra-abdominal tumors, <3 liver lesions that were technically resectable, lack of biliary, and ureteral obstruction; patients in a good general and nutritional state of health, with no severe cardiac, lung, liver, kidney, or neurological condition that could contraindicate the surgery; and patients with no signs of intestinal obstruction and who had an adequate hematologic and hepatic balance. All patients also had to provide specific written informed consent.

The following variables were recorded for each patient:

*Preoperative variables*: age, gender, ECOG performance status, anesthesia risk (according to the American Society of Anesthesiologists (ASA)), body mass index, and preoperative PCI. The preoperative PCI was assessed from the findings of the preoperative abdominopelvic CT; in the event of doubt, the study was completed with PET-TC.

*Intra-operative variables*: PCI, degree of surgical aggression established by the number of peritonectomies and visceral resections required, HIPEC technique used, CC score, and operative time.

*Intra-HIPEC variables* (measured at the end of the cytoreduction, 15 min after starting HIPEC and at the end of the procedure): hemodynamic parameters: O_2_ saturation, systolic and diastolic blood pressure, central venous pressure, heart rate, and maximum inspiratory pressure. Intra-abdominal temperature: recorded at 10, 15, and 30 min after starting the procedure. The hemodynamic parameters were measured via continuous invasive monitoring. The intra-abdominal temperature was recorded with thermal sensors incorporated into the devices to administer the hyperthermia.

The outcome measures were perioperative morbidity and mortality as well as contamination episodes.

### HIPEC procedure

The HIPEC procedure was done following CRS. After the incorporation in our service of HIPEC, we initially started with the Coliseum technique for 2 years, after which we adopted the closed technique, which we continue to use, mainly because of technical improvements and a lower incidence of operating room contamination by chemotherapeutic agents. For us, the technique is more comfortable and safer, avoiding overflow and contamination of operating room personnel, in addition to which its efficacy has been well documented. The cytostatic agent used in both techniques was oxaliplatin at a dose of 460 mg/m^2^ of body surface area, diluted for perfusion in 2 L/m^2^ of glucose solution at 5%, maintaining the perfusion circuit at a constant flow of 0.5–0.7 L/min at 43 °C for 30 min. We used bidirectional chemotherapy to potentiate the intra-peritoneal oxaliplatin by the intravenous administration of folinic acid 20 mg/m^2^, followed by 400 mg/m^2^ of 5-FU in perfusion for 30 min, infused 1 h before the HIPEC. In both techniques, we used three intra-abdominal thermometers (placed in the areas of the pelvis and diaphragm) to monitor the temperature in the peritoneal cavity during perfusion. We also routinely used a probe with an esophageal temperature sensor to monitor the core temperature of the patient. In the HIPEC Coliseum cases, we used a standard technique (described by Sugarbaker). Abdominal wall suspension is achieved with a suture, creating a Coliseum for the instillation of the peritoneal perfusate placing two inflow and two outflow catheters, with a blood recovery device adapted for HIPEC as an infusion pump (Belmont®). The infusion pump forces the perfusate of chemotherapeutic drugs into the abdomen through a Tenckoff catheter and extracts it through the drains, with an approximate flow rate of 1 L/min. A heat exchanger keeps the infused fluid at 43–45 °C so that the intra-peritoneal fluid is maintained at about 41–43 °C. The perfusate first recirculates between the reservoir and the heat exchanger in order for it to reach the suitable temperature. At this point, full circulation of the perfusate into and out of the peritoneal cavity is established until achieving a minimum intra-peritoneal temperature of 41.5 °C. The chemotherapeutic drug is then included in the circuit, at which stage the perfusion timer is started. The perfusion in the cases that underwent closed HIPEC was done in a closed circuit, with placement of two inflow catheters in close proximity to the retroperitoneum and one more superficial outflow catheter. This technique differs from the open technique in that the skin is completely sutured along the laparotomy so that the perfusion is given in a closed circuit. The position of the patient is varied during the perfusion by inclining the operating table in the Trendelenburg and antiTrendelenburg positions and laterally in order to achieve homogeneous distribution of the heat. In comparison with the open technique, a greater volume of fluid is needed in the closed technique to establish the circuit; in addition to which greater intra-abdominal pressures are obtained during the perfusion. In these cases, we used the chemotherapy infusion pump from Evomed®. After the hyperthermic perfusion, the abdomen is again opened and the anastomoses, stomas, and placement of drains are done. Finally, the abdomen is closed definitively in the standard manner.

All the patients in both groups received the same anesthesia protocol.

### Statistical analysis

Initially, a descriptive analysis was made of the study variables, using central tendency and dispersion statistics for the continuous variables. The qualitative variables were studied with frequency distribution tables and their percentages. The comparative analysis of quantitative variables at different times was done with the parametric repeated measure ANOVA for those variables that fulfilled the suppositions of normality and independence, and the Friedman non-parametric test for the variables that did not fulfill either of these suppositions. This analysis was necessary as our objective was to compare observations taken on the same subject over time. To test the suppositions of normality and independence, we used the Shapiro-Wilk test and the Rachas test, respectively. A *p* < 0.05 was considered as significant in all cases. The statistical analysis was done with SPSS v. 17.0 (Chicago, Illinois, USA).

## Results

Our series comprised a total of 30 patients who underwent CRS followed by HIPEC.

### General data

All the patients in both groups shared similar demographic, clinical, and therapeutic features (Table 1). The median age of the patients was 52 years (range 33–66 years), with 16 women and 14 men. All the patients had an ECOG of 0. Analysis of the anesthetic risk (ASA) showed that 20 patients were ASA II, followed by 9 with an ASA of III. Only 1 patient was ASA I. In the series, 17 patients had synchronous peritoneal metastasis, with 13 having metachronous metastasis. In these latter, the median time to appearance of the metastasis was 10 months after surgery for the primary tumor (most in stages pT3N2aM0) and the corresponding adjuvant therapy. The median BMI of the series was 25.12 kg/m^2^ (range 19.7–43.9 kg/m^2^), and the median preoperative PCI was 5.52 (range 0–20). In the Coliseum group, 9 patients received chemotherapy prior to the procedure, while 4 patients in the closed technique group received neoadjuvant therapy.

**Table 1:** Patient characteristics in both groups of treatment, *n* = 30

	Coliseum HIPEC	Closed HIPEC
N Median age	30 52	30 53
Origin of peritoneal metastasis	Colorectal cancer	Colorectal cancer
Timing of peritoneal metástasis		
Synchronous	6	7
Metachronous	8	9
Performance status (ECOG)	0	0
Anesthetic risk (ASA)	ASA I→1	
	ASA II→10	ASA II→10
	ASA III→4	ASA III→5
Median of body mass index (BMI)	25,09	25,15
Median preoperative PCI (peritoneal carcinomatosis index)	5,40	5,57

A total of 30 CRS plus HIPEC procedures were performed. We performed peritonectomy procedures including a large xyphopubic laparotomy, assessing the extension of the peritoneal disease. Staging of the degree of tumor involvement was done with the PCI. All the patients underwent the same surgical procedure, starting with the pelviperitonectomy, resecting the uterus and the adnexa if these were affected, as well as the Douglas pouch. If the rectosigmoid was affected, it was also resected en bloc, with reconstruction of the tract by colorectal anastomosis (or ileorectal in the patients who required total colectomy) with a 29 mm circular stapler. Stomas to protect the anastomosis were done depending on the patient risk factors and the nature of the anastomosis. In the event of implants infiltrating the small intestine and depending on their size, intestinal resections with anastomosis were done or fulguration with the round end of the electro-cauterizer. Cytoreduction of the rest of the peritoneal cavity was then done, systematically performing a complete omentectomy. We always used at least one drain for abdominal aspiration placed in the pelvis, and in those patients who underwent intestinal anastomosis, we placed another aspiration drain nearby.

The median number of peritonectomy and visceral resection procedures required in the HIPEC Coliseum group was 3 (range 0–4), with omentectomy being the most usual, followed by pelviperitonectomy and excision of small bowel implants. The median number of peritonectomy and visceral resection procedures required in the closed HIPEC group was 3 (range 0–4), with pelviperitonectomy and omentectomy being the most usual, followed by excision of small bowel implants and splenectomy. The median intra-operative PCI was 7.58 (range 0–20). After the CRS, 15 patients underwent closed HIPEC and 15 patients received HIPEC with the Coliseum technique. The CC score attained after the CRS procedures was 0 in all cases in both treatment groups. The median number of anastomoses performed in both groups was 2 (range 0–3). The median operative time in both groups was 480 min (range 270–660 min for HIPEC Coliseum and 240–660 min for closed HIPEC). Only one episode of accidental intra-operative contamination from chemotherapeutic agents was recorded in the HIPEC Coliseum group, with no episodes in the closed HIPEC group (difference not statistically significant; *p* = 0.37).

### Intra-HIPEC variables

**Fig. 1:**
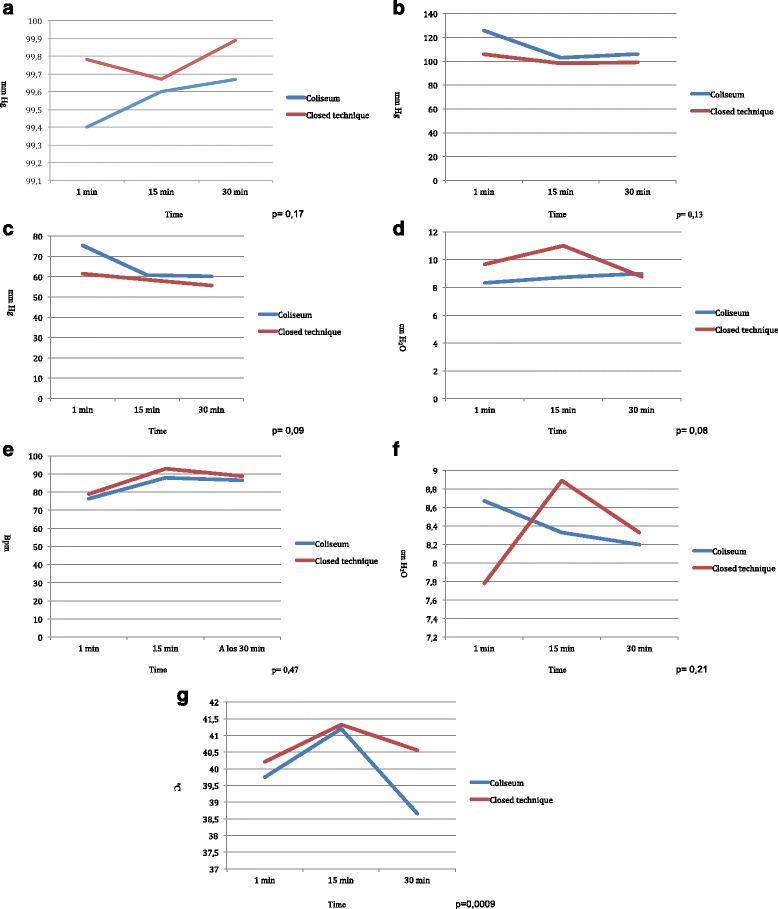
Hemodynamic parameters and abdominal temperature registered during the HIPEC procedure in both groups. **a** O_2_ saturation. The *blue line* corresponds to Coliseum technique. The *red line* corresponds to closed technique. **b** Systolic blood pressure. The *blue line* corresponds to Coliseum technique. The *red line* corresponds to closed technique. **c** Diastolic blood pressure. The *blue line* corresponds to Coliseum technique. The *red line* corresponds to closed technique. **d** Central venous pressure. The *blue line* corresponds to Coliseum technique. The *red line* corresponds to closed technique. **e** Heart rate. The *blue line* corresponds to Coliseum technique. The *red line* corresponds to closed technique. **f** Maximum inspiratory pressure. The *blue line* corresponds to Coliseum technique. The *red line* corresponds to closed technique. **g** Abdominal temperature. The *blue line* corresponds to the Coliseum modality. The *red line* corresponds to closed technique

No statistically significant differences were observed for any of the hemodynamic parameters studied (Fig. 1). The diastolic blood pressure figures varied less during the procedure in the closed technique, being more stable than those with the Coliseum technique. Though the difference was not significant (*p* = 0.09), there was a certain tendency. The central venous pressure figures varied more during the closed technique compared with the Coliseum technique; again not significantly (*p* = 0.08) but with a tendency. Finally, the intra-abdominal temperature remained more stable and homogenous during the closed technique throughout the procedure, varying less than with the Coliseum technique; this difference was statistically significant (*p* = 0.009).

### Morbidity and mortality

All complications arising during the first 30 postoperative days were recorded, classified according to Dindo-Clavien. The incidence of postoperative complications in the HIPEC Coliseum group was 53% (8 patients), all occurring during the early postoperative period (first 15 days). The complications were grade II: urinary fistula requiring percutaneous nephrostomy, nosocomial pneumonia (2 cases), rhabdomyolysis, and acute pulmonary edema; grade IIIA (2 cases): intra-abdominal abscess treated with percutaneous drainage and hepatic abscess that required percutaneous drainage; and grade IIIB (1 case): abdominal sepsis secondary to bacterial translocation that necessitated urgent re-operation. The incidence of complications in the closed HIPEC group was 13% (2 patients). These were grade II (surgical wound infection) and grade IIIB (acute pulmonary edema and acute respiratory failure that required mechanical ventilation).

The differences found concerning morbidity were mainly influenced by the fact that most of the patients in the Coliseum group had received chemotherapy before the procedure (9 cases), whereas only 4 cases who underwent the closed technique had received chemotherapy. Even so, the difference was not statistically significant (*p* = 0.23). Moreover, the complications occurring in the Coliseum group mainly required just conservative management, with just one patient needing urgent re-operation. In the closed technique group, the complications also mainly required just conservative management, with no patient needing urgent re-operation. No death occurred in either of the treatment groups.

## Discussion

The Milan Consensus of 2006 concluded that the most used HIPEC technique was currently the Coliseum technique. This argument was based on the fact that no major advantages have been shown with the open technique versus the closed technique in terms of morbidity or mortality or surgical safety [5].

The present study showed that there are no statistically significant differences in the postoperative morbidity and mortality with the implementation of either technique. While the analysis of the hemodynamic parameters evaluated did not yield any statistically significant differences either, it appears that the closed technique is associated with more stable intra-operative conditions (principally related to the systolic and diastolic blood pressure), exposing the patient to a lesser stress. This proves to be especially helpful in frail patients, with suboptimal preoperative status (older age, comorbidities, and cachexia).

Regarding the hemodynamic monitoring, the present study showed that parameters such as the central venous pressure, heart rate, systolic and diastolic blood pressure, and O_2_ saturation do not differ significantly with the two techniques (*p =* 0.08, *p =* 0.47, *p =* 0.13, *p =* 0.09, and *p =* 0.17, respectively). These findings are in accordance with those of Halkia et al., who reported a retrospective study of 105 patients who underwent CRS and HIPEC, comparing the two techniques. They found no significant differences concerning the hemodynamic parameters between the techniques. A more stable perioperative situation was noted (though not significantly so) with the closed technique versus the Coliseum technique but with very narrow ranges [6]. However, this is not the case with the intra-abdominal temperature. A similar situation was found by Pascual-Ramírez et al. [7], who detected no differences in hemodynamic parameters during CRS and HIPEC when describing the closed technique in ovarian cancer patients. This had also been reported in the study by Desgranges et al. [8].

In our series, we observed differences in heart rate between the open and the closed techniques (increased in the closed technique group). Pascual-Ramírez also reported an increase in heart rate, attributed to increased vasodilatation and relative volume deficit due to heat increase. Indeed, we observed more stable perioperative conditions with the closed technique when we analyzed the systolic and diastolic blood pressure readings, though the differences were not statistically significant to those of the open technique, but with narrower ranges, as found by Halkia et al. For the other study parameters, although greater alterations were detected in the closed HIPEC group, the differences were not statistically significant. However, this observation cannot be evaluated with statistical methods, perhaps owing to the small statistical sample being a limitation of our study.

Schmidt et al., in a retrospective analysis of 78 patients undergoing CRS and HIPEC demonstrated a large intra-operative fluid turnover, an increased airway pressure and central venous pressure (due to the increased intra-abdominal pressure with the closed technique), while the increased body temperature resulted in a mild metabolic acidosis; findings in line with those seen in our study in the closed HIPEC group but with no statistical significance [6, 9].

Facy et al. studied a swine model submitted to closed HIPEC and found that the increase in intra-abdominal pressure produced with the closed technique caused tachycardia, a more aggressive reduction in blood pressure requiring more energetic fluid resuscitation and an increase in ventilation pressure, with stable oxygen saturation, as occurred in our study in the group that underwent surgery with the closed HIPEC technique [6, 10, 11].

Likewise, Cafiero et al., in a study of 15 patients who underwent closed HIPEC, noted that when the intra-abdominal pressure rose there was a reduction in venous return, associated with a drop in abdominal blood volume and a rise in peripheral vascular resistance. On the other hand, they also saw a significant rise in the heart rate, central venous pressure, and pulmonary artery pressure during perfusion (as we also found). These authors concluded that the closed HIPEC procedure is safe for both the patient and the surgical personnel such that the artificial ascites produced does not cause dangerous hemodynamic changes [12].

Schluermann et al. studied 10 patients who underwent closed HIPEC and found similar results concerning the heart rate and central venous pressure. The authors noted that during the closed HIPEC procedure, there was an increase in intra-abdominal pressure associated with a significant reduction in peripheral vascular resistance and an increase in heart rate. These findings could be explained by the appearance of a systemic inflammatory reaction and various mechanisms of physiological adaptation to the increase in body temperature [13].

In our study, we saw that the temperature in the closed HIPEC group was more stable throughout the whole procedure as compared with the Coliseum group. We noted that the intra-abdominal temperature reached the maximum value 15 min after starting the procedure, in both HIPEC techniques. However, there was a progressive fall with effect from 15 min, more pronounced with the Coliseum technique. This latter is because it is more complex to maintain a constant intra-abdominal temperature balance as the procedure is open, in addition to which it requires stirring by the surgeon’s hand, which increases intra-abdominal heat dissipation. Additionally, it is influenced by the low operating room temperature and the measures adopted by the anesthetist to avoid hyperthermia, such as removal of the thermal blanket and fluid and electrolyte replacement with cold fluids during the procedure. Nonetheless, to confront this, we increased the temperature of the inflowing fluids, with the associated risk of potential visceral thermal lesions. In the case of the closed HIPEC technique, while the factor of heat dispersion is not present, the situation is still influenced by other circumstances, such as the low operating room temperature and the measures to avoid hyperthermia.

At the end of the HIPEC procedure, that is at 30 min, temperatures of nearly 39 °C were recorded. This is in line with what has been mentioned earlier; during the procedure the patient experiences a progressive loss of intra-abdominal temperature, despite the hyperthermal administration of fluids, due to heat loss generated by the temperature conditions in the operating room and the measures adopted by the anesthetist to prevent hyperthermia. This loss is more marked with the Coliseum technique than the closed technique. Despite this however, when we noted suboptimal temperatures during the procedure, we attempted to compensate by raising the temperature of the inflowing fluid. The differences found in intra-abdominal temperature were statistically significant (*p =* 0.0009).

Some studies have shown that the closed technique does not guarantee thermal homogeneity. Ortega-Deballon et al. undertook a study in an animal model to compare the open and closed techniques in nine pigs with the aim of analyzing the thermal homogeneity and diffusion of the chemotherapeutic agent. Hyperthermia was achieved in both study arms, but it was not homogeneous in the closed technique group. This is due to the proximity of the inflow catheters to the diaphragm in the absence of stirring in the closed technique. Nevertheless, the problem concerning stirring the fluid inside the abdominal cavity when using the closed technique, and thus maintaining thermal homogeneity with this technique, could be solved by undertaking a closed HIPEC using a laparoscopic approach, thus enabling uniform distribution of temperature and cytotoxic agents. This constitutes a future line of research to improve heat distribution during HIPEC [14]. The high dissipation of heat in the open technique could explain the need for greater flow when a heat pump is used [4, 6].

Padmanabhan et al. found difficulties maintaining the intra-abdominal temperature with the Coliseum technique, which is why they then used the closed technique in the other cases. They based this on the fact that one of the great advantages of the closed technique is the minimal exposure of operating room personnel to the chemotherapeutic agent as well as the better maintenance of the intra-abdominal temperature [4, 15–19]. Our findings agree with those seen by Padmanabhan et al. However, the beneficial effect of the high intra-abdominal pressure that can theoretically be achieved with the closed technique may be offset by the existence of preferential circuits with under-treated areas of the abdominal cavity during HIPEC [4, 20].

## Conclusions

Both the open and the closed abdomen techniques are safe and efficient methods of HIPEC delivery in the treatment of peritoneal carcinomatosis.

In our series use of the closed technique was associated with fewer hemodynamic alterations relating to the systolic and diastolic blood pressure readings in comparison with the Coliseum technique, though the differences were not statistically significant. Nevertheless, the closed technique resulted in less variation in intra-abdominal temperature, which was significantly more stable throughout the procedure.

## Abbreviations

BMI: Body mass index
CC score: Completeness of cytoreduction score
CRS: Cytoreductive surgery
ECOG: Eastern Cooperative Oncology Group
HIPEC: Intraoperative hyperthermic intraperitoneal chemotherapy
O_2_: Oxygen
PCI: Peritoneal carcinomatosis index

## References

[1] Yonemura Y, Canbay E, Ishibashi H (2013). Prognostic factors of peritoneal metastases from colorectal cancer following cytoreductive surgery and perioperative chemotherapy. Sci World J.

[2] Di Giorgio, A; Pinto, E. Treatment of peritoneal surface malignancies. Cap 3. Springer.

[3] Virzi, S; Iusco, D; Bonomi, S; et al. Hyperthermic intraperitoneal chemotherapy (HIPEC) techniques (Cap 10). Treatment of Peritoneal Surface Malignancies. Springer.

[4] Ortega-Deballon P, Facy O, Jambet S (2010). Which method to deliver hyperthermic intraperitoneal chemotherapy with oxaliplatin? An experimental comparison of open and closed technique. Ann Surg Oncol.

[5] González Moreno S, González Bayon L, Ortega Pérez G (2010). Hyperthermic intraperitoneal chemotherapy: rationale and technique. World J Gastrointest Oncol.

[6] Halkia, A; Tsochrinis, A; Vassiliadou, DT; et al. Peritoneal carcinomatosis: intraoperative parameters in open (coliseum) versus closed abdomen HIPEC. Int J Surg Oncol. 2015;2015:610597. 10.1155/2015/610597 PMC4345051 25785194

[7] Pascual-Ramírez J, Sánchez García S, de la Herrán GRF (2014). Security and efficiency of a closed-system, turbulent flow circuit for hyperthermic intraperitoneal chemotherapy after cytoreductive ovarian surgery: perioperative outputs. Arch Gynecol Obstet.

[8] Desgranges FP, Steghens A, Rosay H (2012). Epidural analgesia for surgical treatment of peritoneal carcinomatosis: a risky technique?. Ann Fr Anesth Reanim.

[9] Schmidt C, Creutzenberg M, Piso P (2008). Perioperative anaesthetic management of cytoreductive surgery with hyperthermic intraperitoneal chemotherapy. Anaesthesia.

[10] Facy O, Al Samman S, Magnin G (2012). High pressure enhances the effect of hyperthermia in intraperitoneal chemotherapy with oxaliplatin: an experimental study. Ann Surg.

[11] Facy O, Combier C, Pussier M (2015). High pressure does not counterbalance the advantages of open techniques over closed techniques during heated intraperitoneal chemotherapy with oxaliplatin. Surgery.

[12] Cafiero T, Di Iorio C, Di Minno RM (2006). Non-invasive cardiac monitoring by aortic blood flow determination in patients undergoing hyperthermic intraperitoneal intraoperative chemotherapy. Minerva Anestesiol.

[13] Schluermann CN, Hoeppner J, Benk C (2016). Intraabdominal pressure, Cardiac Index and vascular resistance during hyperthermic intraperitoneal chemotherapy: a prospective observational study. Minerva Anestesiol.

[14] Lotti M (2016). Laparoscopic HIPEC: a bridge between open and closed-techniques. J Min Access Surg.

[15] Padmanabhan N, Kumar BR, Pookunju AP (2015). Preliminary experience and morbidity analysis of cytoreductive surgery with hyperthermic intraperitoneal chemotherapy (CRS/HIPEC) from a tertiary cancer center in India. J Clin Diagn Res.

[16] Loggie BW, Fleming RA, McQuellon RP (2000). Cytoreductive surgery with intraperitoneal hyperthermic chemotherapy for disseminated peritoneal cancer of gastrointestinal origin. Am Surg.

[17] Garofalo A, Valle M, García J (2006). Laparoscopic intraperitoneal hyperthermic chemotherapy for palliation of debilitating malignant ascites. Eur J Surg Oncol.

[18] Gesson-Paute, A; Ferron, G; Thomas, F et al. Pharmacokinetics of oxaliplatn during open versus laparoscopically assisted heated intraoperative intraperitoneal chemotherapy (HIPEC): an experimental study. Ann Surg Oncol. 2008;15(1):339-44. 10.1245/s10434-007-9571-9 17943387

[19] Elias D, Bonnay M, Puizillou M (2002). Heated intra-operative intraperitoneal oxaliplatin after complete resection of peritoneal carcinomatosis: pharmacokinetics and tissue distribution. Ann Oncol.

[20] Sarnaik AA, Sussman JJ, Ahmad SA (2007). Technology for the delivery of hyperthermic intraoperative intraperitoneal chemotherapy: a survey of techniques. Recent Results Cancer Res.

